# A systematic review of EEG-based machine learning classifications for obsessive-compulsive disorder: current status and future directions

**DOI:** 10.1186/s12888-025-07296-z

**Published:** 2025-09-09

**Authors:** Mahdi Naderi, Amir Jahanian-Najafabadi

**Affiliations:** 1 Department of Cognitive Sciences, Faculty of Psychology and Educational Sciences, Shiraz University, Shiraz, Iran; 2 Department of Cognitive Neuroscience, Faculty of Biology, Bielefeld University, Bielefeld, Germany

**Keywords:** Obsessive-compulsive disorder, Machine learning, Deep learning, Electroencephalography, Random forests, Convolutional neural networks

## Abstract

Obsessive–compulsive disorder (OCD) is a chronic and disabling condition affecting approximately 3.5% of the global population, with diagnosis on average delayed by 7.1 years or often confounded with other psychiatric disorders. Advances in electroencephalography (EEG) analysis using machine learning hold promise for the development of OCD-specific biological markers. This systematic review aims to evaluate studies that classify individuals with OCD from other groups based on EEG data. Following PRISMA guidelines, we searched the Web of Science, Scopus, PubMed, and IEEE databases through February 2025; of 42 screened studies, 11 met inclusion criteria for final analysis. Data were extracted across four domains: general information, population characteristics, EEG features, and machine learning features. Results revealed extensive heterogeneity in study populations, associated symptoms, EEG preprocessing methods, validation strategies, and reporting of model accuracy, underscoring the need for harmonized standards. Notably, only a few studies provided statistical interpretation of their models. None of reviewed studies employed modern interpretability techniques such as SHAP or LIME methods that, beyond reducing “black-box” opacity, can inform optimal electrode placement for neurofeedback or transcranial electrical stimulation. Many studies were constrained by cultural limitations, small sample sizes and lack of demographic information e.g., age, gender, medication. This work represents the first systematic review of EEG-ML classification studies in OCD and emphasizes the urgent need for methodological standardization in this emerging field.

## Introduction

Obsessive-Compulsive Disorder (OCD) is a common and long-lasting mental health condition that affects a person’s social life, family, and society [1]. About 60% of people with OCD experience chronic symptoms in their lives [2]. OCD affects around 3.5% of people, with women (5.4%) more likely to have it than men (1.7%) [2]. A study found that half of OCD symptoms start by age 18, and 70% start before age 20. This shows why looking for early signs in teenagers is important [2]. OCD greatly reduces a person’s quality of life and work performance [2].

More than half of patients diagnosed with OCD have serious problems in family relationships and daily activities because of the symptoms [3]. The costs of OCD both direct e.g., cost of treatment and indirect such as lost work and associated income are very high [3]. Because of this, the World Health Organization (WHO) listed OCD as one of the most disabling mental disorders which extremely affect life of individuals who suffer from OCD sysmptoms and their families [4]. Even though OCD is serious psychiatric condition, many people do not get diagnosed or treated early in life. On average, it takes 7.1 years from the onset of symptoms to the diagnosis stage [5]. Reasons for this delay include social stigma, limited healthcare access, and financial problems [6]. Additionally, OCD can be misdiagnosed by specialists due to the similarity and overlaps of symptoms with other psychiatric disorders, or potentially due to the feeling of shame in patients [7]. A study found that 33.8% of African-American patients with OCD were not diagnosed correctly and with precision. This may be because common tools for diagnosis do not work well for this group [8].

For treatment-resistant OCD, methods like antipsychotic drugs, deep brain stimulation, and neurosurgical interventions can help [9]showing the importance of brain-focused treatments. EEG with high temporal resolution at the millisecond scale is beneficial because it can provide biological markers and show how the brain is affected both anatomically and functionally [10]. Studies using EEG have found irregularities in the frontotemporal brain areas, especially in women and those who respond to treatment [11]. EEG is also affordable [12, 13]easy to use, and helpful in emergency conditions [13]. Application of machine learning techniques in classification of OCD compared to healthy individuals and to distinguish with other psychiatric conditions suggests a new avenue to improve the diagnostic tools for mental disorders e.g., OCD.

With advanced computational modelings and machine learning techniques we are able to detect unusual/abnormal brain patterns and help diagnose mental disorders [14, 15]. These methods can also suggest personalized treatments based on brain activity [16, 17]. However, challenges like data standardization and model accuracy still exist [15, 16, 18]. There have been several computational studies in recent years that have aimed to either distinguish healthy from other neuropsychiatric and neurodevelopmental disorders, e.g., ADHD [19]; Autism Spectrum Disorder [20, 21]; MDD [22–24] or for time-series forecasting in order to predict EEG signals [25].

In order to apply machine learning models in practice, their interpretability is of great importance. Modern interpretability techniques such as SHAP (SHapley Additive exPlanations) and LIME (Local Interpretable Model-agnostic Explanations), by identifying the relative importance of features in the model’s prediction, can provide researchers and clinicians with a better understanding of the contribution of each variable for instance, the relative importance of EEG electrodes within different frequency bands. LIME generates synthetic samples around the target instance and fits a simple local linear model to the outputs of the main model to extract the local influence of each feature, while SHAP calculates the fair contribution of each feature across all possible feature combinations, assigning importance values that are disentangled and independent from other features [26–28]. For example, recent studies in the fields of EEG in response to treatment [29]classification of stroke [30]and diagnosis of depression [31] have effectively utilized SHAP and LIME to analyze and determine the importance of EEG features. However, to our knowledge, this is the first systematic review focused on EEG-based machine learning for classification of OCD compared to healthy(other) group(s). In this research, we examined recent progress and highlights the need for improvement of research methods and filling knowledge gaps.

## Methods

### Search strategy

**Fig. 1:**
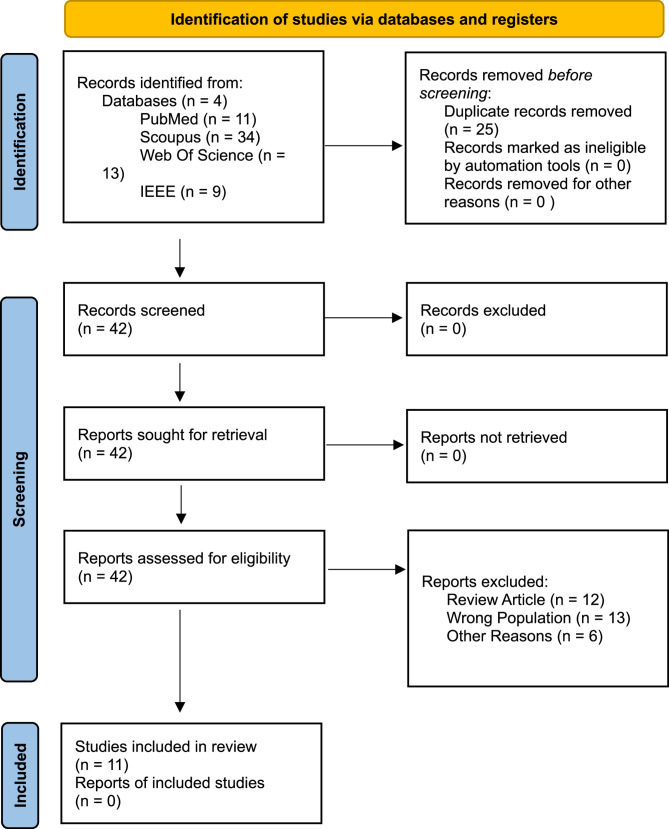
PRISMA flowchart for the selection of studies

Following PRISMA guidelines, a systematic search was conducted in PubMed, Scopus, IEEE, and Web of Science databases from its research inception to 6 February 2025. After the search, 67 articles were found (cf., Fig. 1). These articles were imported into EndNote software and duplicates were removed. After removing duplicates, 42 articles remained. These 42 articles were extracted and imported into Rayyan and we reviewed independently to include or exclude articles based on the inclusion/exclusion criteria. If there was a disagreement, we further evaluated and discussed until we agreed. Finally, 11 articles were included in this study. The accepted articles were entered into an Excel file with four main areas:

- General information (Table 1).

**Table 1:** General information of included studies

Study	Journal Name	Disorders Studied	Ethics Committee Approval or Informed Consent Process	Country /Region
Ahmed et al., 2024	IBRO Neuroscience Reports	SCZ; MDD; BD; PD; SAD; OCD; AUD; BA; PTSD; ASD; AD	_	Pakistan Turkiye Finland
Basak et al., 2023	2023 IEEE International Conference on Computer Vision and Machine Intelligence (CVMI)	SCZ; Anxiety Disorder; OCD; SUD; PTSD	_	India
Emre et al., 2023	International Journal of Medical Informatics	BD; ADHD; MDD; OCD; PTSD; OUD; SCZ; HC	Uskudar university committee: JUNE2021-40/1,351,342	Turkiye
Erguzel et al., 2015	Neurocomputing	TTM; OCD	_	Turkiye
Farhad et al., 2024	Clinical EEG and Neuroscience	OCD; HC	_	Turkiye
Gour et al., 2023	Brain Informatics	MDD; ADHD; SMC; OCD; HC	_	United Arab Emirates
Ma et al., 2025	Journal of Psychiatric Research	OCD with 2 levels of anxiety; HC	Central South University Ethics Committee: Approval granted.	China
Mukherjee et al., 2024	2024 International Conference on Intelligent Algorithms for Computational Intelligence Systems (IACIS)	Addiction disorder; Trauma & stress related; Mood disorder; SCZ; OCD; HC	_	India
Park et al., 2021	Frontiers in Psychiatry	SCZ; MDD; BD; PD; SAD; OCD; AUD; Behavioral Addictions; PTSD; ASD; adjustment disorder	IRB Approval: 20-2019-16 (consent waived for retrospective study)	Korea
Ren et al., 2024	BMC Psychiatry	OCD	For Dataset 2: Xinxiang Medical University Ethics Committee: Ethics approval obtained	China
Verma et al., 2024	2024 International Conference on Communication, Computer Sciences and Engineering (IC3SE)	anxiety disorder; addictive disorder; Trauma and stress-related disorders; Mood disorders; OCD; SCZ; HC	_	India
- Population (Table 2).

**Table 2:** Population characteristics and clinical demographics

Study	OCD Symptom Severity	How was OCD diagnosed??	Subtype of OCD	Comorbidities	Number of Population	Treatment History
Ahmed et al., 2024					total 945 (850 patient 95 HC)	
Basak et al., 2023					total 1330	
Emre et al., 2023		psychiatrist diagnosis			total 550 (HC 84 OCD 34)	
Erguzel et al., 2015	Average 23/6 in Y-BOCS	psychiatrist diagnosis		not other disorder	39 TTM 40 OCD	under drug not mood and lithium
Farhad et al., 2024					52 HC 30 man 22 woman 86 OCD 42 man 44 woman	
Gour et al., 2023						
Ma et al., 2025	OCD: Y-BOCS > 16 (ave = 23/18 SD = 4/19) anxiety: HAMA (ave = 16/29 SD = 7/41)	MINI 4.0.2 Y-BOCS HAMA	OCD + Moderate anxiety OCD + mild anxiety	Anxiety levels	48 OCD (21 man, 27 woman) 33 over Moderate anxiety 15 mild anxity	under drug: SSRI treatment (*n* = 19) Of these 19: 8 also used second-generation antipsychotics
Mukherjee et al., 2024					46 OCD 95 HC total 945	
Park et al., 2021		psychiatrist MINI			46 OCD (38 man 8 woman) 95 HC total: 945	
Ren et al., 2024	Y-BOCS > 16	psychiatrist Y-BOCS			48 OCD 48 HC (Database1: 32 OCD 33 HC Database: 16 OCD 15 HC)	
Verma et al., 2024					850 mental disorders 95 HC
- EEG features (Table 3).

**Table 3:** EEG recording protocols and feature extraction methods

Study	OCD vs.	Data colle- -ction?	Preprocessing steps	eeg montage	Frequency bands analyzed	frequency sampling	Eyes/Rest-Task-related	Feature extraction methods	Feature selection
Ahmed et al., 2024	Other Disorder	No	0.1–100 on-line filter > one-hot coding discretness of future > standardization	10_20 19CH Ref: mastoid	delta theta alpha beta highbeta gamma	500–1000 hz	Close - Rest	PSD FC FFT	
Basak et al., 2023	Other Disorder	No	downsampling > 19CH > FFT > PSD > FC > moving average	10_20			Open-Rest	PSO PSD FC	PSO
Emre et al., 2023	HC & Other Disorder	Yes	normalized (min-max, Z score, log 10) > undersampling and oversampling > band-pass 0.3–70 hz > artifact remove	10_20 Ref: ear A1-A2	delta theta alpha beta		Open-Rest	FFT	
Erguzel et al., 2015	Other Disorder	Yes	Bandpass Filter 0/15-30hz > Manual Artifact Rejection > FFT	10_20 Ref: 2 ears	delta theta alpha beta	250 hz	Close - Rest	FFT Chordance	IACO
Farhad et al., 2024	HC	Yes	filter 1-10hz > ICA >.edf fotmat	10_20 ground: between FZ & Fpz	raw signal	125 hz	Close - Rest	raw signal	
Gour et al., 2023	HC & Other Disorder	No	bandpass filter 50 hz > down-sampling to 100 hz > 3 s trimmed both ends > normalize to Zscore > Split into 10 s epochs, 2 s overlap	10_10	raw signal	500 hz	Close - rest & Open – dot red	raw signal	CCE, WCCE, FALCON
Ma et al., 2025	OCD + Mild/ Moderate & anxiety HC	Yes	bandpass 1-40hz > ICA > refrencing to average > delete some part of + − 100µV	10_20 Ref: average CH		256 hz	Close – Rest	PSD, FC, FFT, PSO, Chordance, Raw, Microstate (metrics), Hurst, Markov, Stacked AE, Entropy	Microstate, Boruta, FW(MRMR), GA, SVM-RFE
Mukherjee et al., 2024	HC & Other Disorder	No		10_20	delta theta alpha beta		NA	PSD Stacked Autoencoder	Stacked Autoencoder
Park et al., 2021	HC & Other Disorder	Yes	online filter 0/1-100hz	10_20 ref: mastoid	delta theta alpha beta highbeta gamma	125 hz	Close - Rest	PSD FC FFT	Elastic Net
Ren et al., 2024	HC	Database 1: No Database 2: Yes	Bandpass filter 2–20 Hz > Downsampling to 250 Hz > ICA > Data segmented into 2-sec epochs > First 90 s of data selected for microstate analysis	analysis: 19 commen CH	microstate analysis	analysis: 250	Close - Rest	microstate analysis (sampling entropy, LZC, Hurst)	
Verma et al., 2024	HC	Yes	standard scaler > remove artifacts > Data aygmentation		delta theta alpha beta highbeta gamma		Open-Rest	PSD FC
- Machine learning features (Table 4).

**Table 4:** Machine learning methodologies and performance metrics

Study	Machin learning algoritm	Machine learning accuracy	Validation method	Class imbalance handling	Model interpretability methods	Model interpretabili output
Ahmed et al., 2024	ANN KNN LSTM Bi LSTM CNN-LSTM	accuracy OCD: ANN: 96.83 KNN: 94.70 LSTM: 93.65 Bi LSTM: 93.12 CNN-LSTM: 92.06 accuracy overall: ANN:87% KNN:85% LSTM:86% Bi-LSTM:87% CNN-LSTM:86%	cross-validation in 100 loop			
Basak et al., 2023	LSTM	Precision OCD:95 Recollection OCD:100 F1-Score OCD:97 overall: Validation Accuracy: 89.85 Training Accuracy:99.15 Weighted Avg F1-Score:90	train - test 1064 train 266 test	SMOTE		
Emre et al., 2023	C5.0 RF SVM ANN	Model C5.0 (imbalanced dataset): Overall accuracy: 84.1 Sensitivity: 42.9 Specificity: 98.3 F-score: 50.0 Balanced accuracy: 70.6 Model SVM_radial (oversampling dataset): Overall accuracy: 84.1 Sensitivity: 85.7 Specificity: 95.0 F-score: 63.2 Balanced accuracy: 90.3 Model RF (undersampling dataset): Overall accuracy: 76.2 Sensitivity: 85.7 Specificity: 93.3 F-score: 57.1 Balanced accuracy: 89.5	5-fold repeats 3 time	undersampling oversampling		
Erguzel et al., 2015	ANN KNN SVM NB	ANN OCD: 63.29 KNN OCD: 56.96 SVM OCD: 67.08 NB OCD: 56.96 IACO-SVM OCD: 81.04	10-fold cross-validation	they are balance		
Farhad et al., 2024	1DCNN-LSTM 1DCNN-GRN	1DCNN-LSTM Cross-Validation OCD: 90.88 1DCNN-LSTM External Validation OCD: 75 1DCNN-GRN Cross-Validation OCD: 85.91 1DCNN-GRN External Validation OCD: 75	5-fold		Gini impurity XGBoost Permutation Drop column	important electrods: O2, T5, T6, F7
Gour et al., 2023	Transformer with 4 block encoders and 8 attention head	accuracy overall: Transformer + CCE (EO): 63.21% Transformer + CCE (EC): 61.74% Transformer + FL (EC): 68.49% Transformer + FL (EO): 68.49%	test - train majority voting	WCCE Focal Loss		
Ma et al., 2025	SVM XGBoost RF	Classification Accuracy of OCD and HC: Random Forest Val AUC: 67.10% (all features), 68.47% (GA), 67.27% (FW), 69.79% (Boruta), 68.64% (SVMRFE) Test AUC: 68.38% (all features), 68.75% (GA), 69.65% (FW), 68.22% (Boruta), 69.15% (SVMRFE) Linear SVM Val AUC: 68.25% (all features), 64.27% (GA), 66.26% (FW), 66.48% (Boruta), 68.78% (SVMRFE) Test AUC: 68.55% (all features), 63.66% (GA), 64.83% (FW), 64.92% (Boruta), 68.40% (SVMRFE) Poly SVM Val AUC: 61.99% (all features), 65.40% (GA), 63.09% (FW), 65.67% (Boruta), 65.54% (SVMRFE) Test AUC: 59.92% (all features), 61.45% (GA), 61.41% (FW), 65.70% (Boruta), 64.91% (SVMRFE) RBF SVM Val AUC: 69.54% (all features), 72.75% (GA), 69.84% (FW), 69.20% (Boruta), 71.22% (SVMRFE) Test AUC: 68.15% (all features), 68.03% (GA), 70.43% (FW), 68.74% (Boruta), 69.42% (SVMRFE) XGBoost Val AUC: 66.43% (all features), 68.16% (GA), 66.90% (FW), 69.01% (Boruta), 68.27% (SVMRFE) Test AUC: 68.70% (all features), 68.49% (GA), 69.28% (FW), 67.33% (Boruta), 68.83% (SVMRFE) Classification Accuracy of OCD Subgroups: Random Forest Val AUC: 71.28% (all features), 73.37% (GA), 74.04% (FW), 74.11% (Boruta), 71.38% (SVMRFE) Test AUC: 71.61% (all features), 73.81% (GA), 73.03% (FW), 73.52% (Boruta), 71.25% (SVMRFE) Linear SVM Val AUC: 70.83% (all features), 71.10% (GA), 71.22% (FW), 71.74% (Boruta), 72.27% (SVMRFE) Test AUC: 66.22% (all features), 66.46% (GA), 66.25% (FW), 68.29% (Boruta), 68.03% (SVMRFE) Poly SVM Val AUC: 68.04% (all features), 69.78% (GA), 69.82% (FW), 69.42% (Boruta), 69.81% (SVMRFE) Test AUC: 61.51% (all features), 65.60% (GA), 63.37% (FW), 62.97% (Boruta), 62.46% (SVMRFE) RBF SVM Val AUC: 72.21% (all features), 72.50% (GA), 71.91% (FW), 72.57% (Boruta), 72.17% (SVMRFE) Test AUC: 66.92% (all features), 68.88% (GA), 67.89% (FW), 68.86% (Boruta), 68.05% (SVMRFE) XGBoost Val AUC: 69.59% (all features), 71.00% (GA), 72.86% (FW), 72.69% (Boruta), 69.84% (SVMRFE) Test AUC: 74.15% (all features), 77.13% (GA), 72.89% (FW), 72.16% (Boruta), 70.68% (SVMRFE)	test – train	SMOTE	using statistic	The occurrence probability of EEG microstate C shows a significant negative correlation with HAMA scores. EEG microstate feature indices are significantly associated with the severity of obsessive thoughts and anxiety symptoms.
Mukherjee et al., 2024	PsyNet = Autoencoder + PLSTM	PsyNet OCD accuracy :100 overall accuracy: 95.97	10-fold cross-validation		attenstion mechanism	
Park et al., 2021	Elastic Net SVM RF	OCD in FC of Gamma Elastic Net: 74.52 Elastic Net overall: 87.59 SVM overall: 86.02 RF overall: 87.18	10-fold cross-validation		using statistic	Decrease: fp1 fp2 Decrease: fp1 f3 Decrease: f3 fz Increase: c4 p4
Ren et al., 2024	SVM LR GNB	set1 accuracy (time futures): SVM: 75.86 LR: 72.41 GNB: 75.86 set2 accuracy (nonlinear futures): SVM: 80 LR: 85 GNB: 80	5-fold cross-validation	they are balance	using statistic	The presence of differences in the topography among different OCD subgroups.
Verma et al., 2024	CNN-GRU	OCD accuracy in FC of BETA: 90 overall: 90–96	train – test	augmentation

In the next step, the extracted data were interpreted qualitatively. A quantitative meta-analysis was not performed due to substantial heterogeneity across studies. Specifically, the sample sizes, and that the reviewed studies employed a wide variety of EEG preprocessing pipelines, including different filtering, artifact removal, and feature extraction techniques. In addition, the machine learning models varied considerably, encompassing SVMs, Random Forests, Convolutional Neural Networks (CNNs), and ensemble methods, with diverse validation strategies such as 10-fold cross-validation, leave-one-out, and hold-out methods. These methodological differences, along with inconsistent reporting of effect sizes and outcomes, precluded meaningful statistical synthesis. While no formal heterogeneity statistics could be calculated due to insufficient comparable data, the degree of variation in design and analysis across studies supports the decision to pursue a qualitative synthesis.

### Search terms

(“EEG” OR “electroencephalography” OR “resting state EEG” OR “resting-state EEG”) AND (“machine learning” OR “artificial intelligence” OR “deep learning”) AND (“Obsessive compulsive disorder” OR “OCD” OR “Obsessive-Compulsive”) AND (“classification” OR “diagnosis” OR “pattern recognition” OR “taxonomy” OR “Convolutional Neural Network” OR “Random Forest” OR “Short Long Term Memory” OR “LSTM” OR “Computational Modeling” OR “Early diagnosis”).

### Inclusion/Exclusion criteria

Inclusion criteria:

- Studies with OCD population.
- Use of machine learning techniques applied to OCD-related EEG data.
- Peer-reviewed.
- Full-text available.
- Published in English.
- Publication date from database inception up to February 2025.

Exclusion criteria:

- Studies not directly applying machine learning for classification.
- Studies not focusing on OCD patients.
- Studies not using EEG for OCD-related classification.
- Non-English articles.
- Articles that are not original research.
- Case reports.

### Quality of studies

To check the risk of bias in the studies we included, we used a changed version of the QUADAS-2 tool. This tool was first made for medical diagnosis studies, but we used it for three main reasons. First, we could change the questions to match ML-EEG studies, but still keep the four main parts: how patients were chosen, how the test was done, how OCD was diagnosed, and how data was used over time. Second, these parts match the important steps in EEG and machine learning research. Third, there is no special tool made for EEG-ML studies in psychiatry yet. So, QUADAS-2 was a good and flexible option. Two reviewers checked the bias separately, and they talked to agree if there were any differences. Table 5 present QUADAS-2 criteria employd for EEG-ML studies.

**Table 5:** Adapted QUADAS-2 criteria for EEG-ML studies

Domain	Adapted Signaling Question	Low Risk	Unclear Risk	High Risk
Patient Selection	Was patient selection done so that it was not affected by comorbidities or medication?	The study explicitly excluded or separately analyzed patients with comorbidities or medication.	The study did not report whether patients had comorbidities or were under medication.	The study included patients with heterogeneous comorbidities or medication in the same analysis group.
Index Test	Was data splitting into training and test sets done randomly?	According to codes or text, it is clear that the individuals were randomly divided into training data and testing data.	The article and its code do not specify whether the data split into training and testing sets was done randomly.	The article indicates that the data split into training and testing sets was performed non-randomly and with bias.
Reference Standard	Was OCD diagnosed using standard criteria such as DSM-5 or ICD-10?	Diagnosis was performed using DSM-5, ICD-10, or validated instruments (e.g., MINI, Y-BOCS).	Diagnosis method was not mentioned or not clearly described.	Diagnosis was based on non-validated tools or unclear clinical criteria.
Flow and Timing	Was the order of giving train and test data to machine learning correct to avoid data leakage?	Clear evidence that training data was kept separate from test data (no leakage).	Unclear whether data leakage was prevented during training/testing.	Evidence that test data was used during training or feature selection (leakage confirmed).

### Risk of bias

The highest risk of bias was identified in the patient selection domain, while the lowest risk was observed in the flow and timing domain. Many studies appeared to prioritize an engineering-focused approach, emphasizing model development over detailed descriptions of patient populations. Several studies did not clearly report how OCD patients were screened. Random data splitting was confirmed in only five studies, while the remaining six did not provide sufficient information (cf., Table 6). Studies reporting very high accuracy such as Mukherjee et al. (2024) with 100% accuracy which also exhibited the highest risks of unclear bias. Conversely, studies with lower bias levels such as Erguzel et al. (2015) reporting 57–81% accuracy tended to align more closely with clinical reality. These findings suggest that minimizing bias should take precedence over simply maximizing accuracy.

**Table 6:** Risk of bias assessment using modified QUADAS-2 criteria

	PATIENT SELECTION	INDEX TEST	REFERENCE STANDARD	FLOW AND TIMING
Ahmed et al., 2024	Unclear	Low	Unclear	Low
Basak et al., 2023	Low	Low	Unclear	Low
Emre et al., 2023	Low	Unclear	Low	Low
Erguzel et al., 2015	Low	Low	Low	Unclear
Farhad et al., 2024	Unclear	Low	Unclear	Low
Gour et al., 2023	Unclear	Unclear	Unclear	Low
Ma et al., 2025	High	Unclear	Low	Low
Mukherjee et al., 2024	Unclear	Unclear	Unclear	Low
Park et al., 2021	High	Low	Low	Low
Ren et al., 2024	High	Unclear	Unclear	Low
Verma et al., 2024	Unclear	Unclear	Unclear	Unclear

## Result

In this systematic review, we examined studies that use EEG and machine learning to classify patients with OCD from other clinical conditions. As this is a relatively new emerging field, this review serves as a foundational step in identifying current gaps and offering suggestions for future EEG-ML research and reporting. Narrative synthesis of studies is shown in four areas:

- General study information (Table 1).
- Test population (Table 2).
- EEG features (Table 3).
- Machine learning features (Table 4).

This study follows PRISMA diagram to improve quality and transparency.

### Emerging field

The application of EEG and machine learning in psychiatric diagnosis, particularly for OCD is a rapidly growing field. Prior to 2020, only one relevant study had been published. However, in the past five years, the number has increased to 11 studies, highlighting a significant rise in research and potentially clinical interest. This growth is largely driven by recent advancements in deep learning and machine learning techniques and better availability of datasets [32–34]. Despite this progress, substantial research gaps remain, underscoring the need for methodological and clinical refinement. The relatively small number of studies also emphasizes the nascent stage of this research domain.

### Geographical spread and generalization issues

**Fig. 2:**
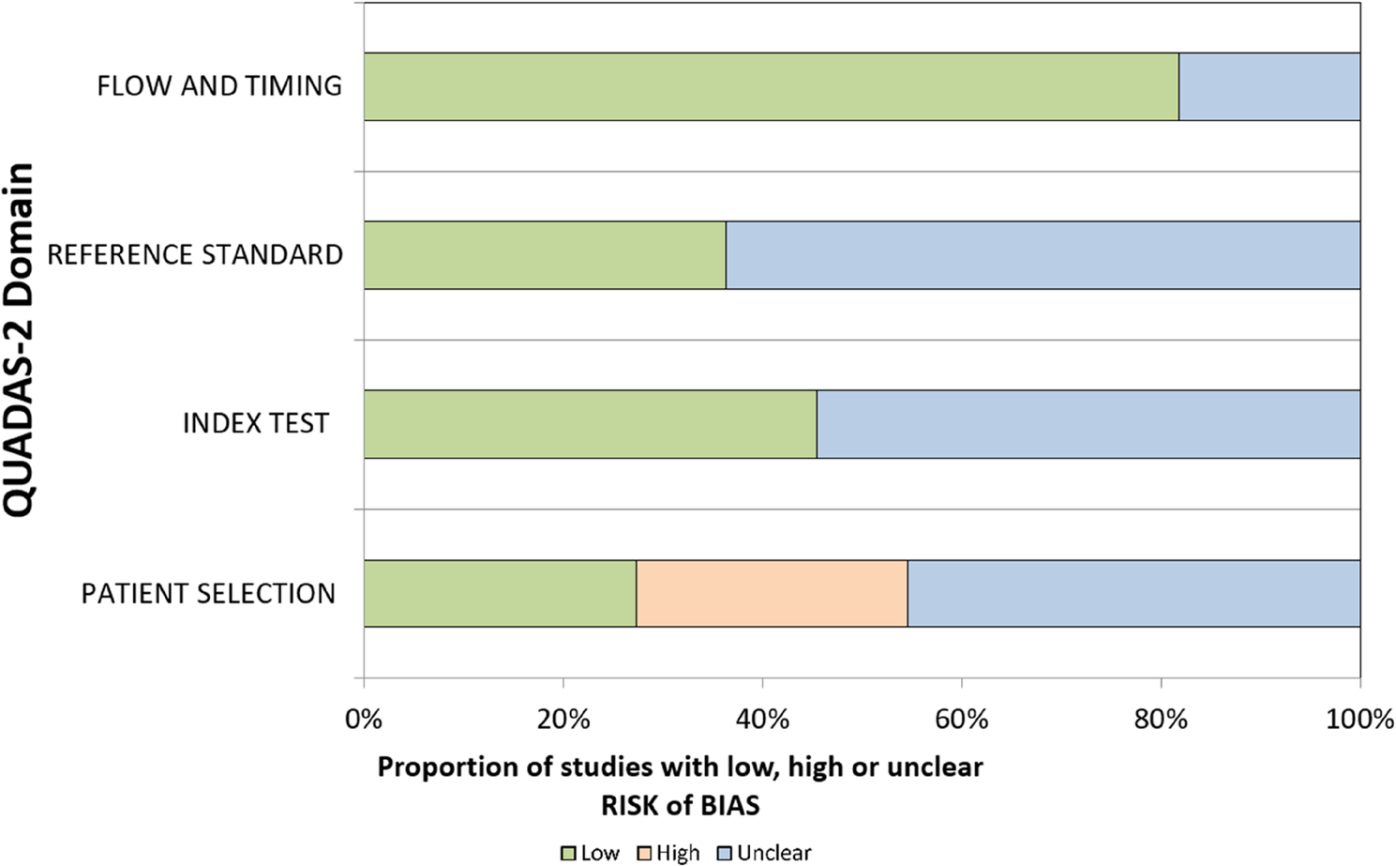
Visual Summary of Bias Risk Distribution

Among the 11 studies analyzed, 10 originated from Asian countries, with only one involving a Europe–Asia collaboration (cf., Fig. 2). This limited geographic distribution raises concerns about the generalizability of findings to non-Asian or Western populations. However, the use of publicly available datasets may mitigate some of this bias. Of the included studies, 36% (*n* = 4) used public datasets, 54% (*n* = 6) relied on original data collection, and 9% (*n* = 1) employed a combination of public and private data. The growing use of shared datasets represents a promising avenue for enhancing reproducibility and cross-cultural applicability in future research.

### Different definitions of OCD

Approximately 54% of the reviewed studies did not report how OCD patients were screened, resulting in an “unclear” risk of bias rating for the reference standard domain in the QUADAS-2 assessment (cf., Table 6). Only 18% (*n* = 2) explicitly stated that psychiatrists conducted the screening, while 9% (*n* = 1) relied on self-report questionnaires. Of the six studies that omitted screening details, four used public datasets and two used privately collected data. Although public datasets improve accessibility and facilitate research, they often lack critical demographic and diagnostic information, which may introduce bias. These findings highlight the need for standardized, publicly available EEG datasets with clear documentation and clinical validation such as Deutsche Forschungsgemeinschaft (DFG)- based guidelines.

We identified a significant research gap in the reviewed studies which nearly all studies have overlooked DSM-4/DSM-5 OCD subtypes (such as contamination and symmetry) in EEG-based machine learning analyses. Treating OCD as a homogeneous disorder risks oversimplifying its neural underpinnings. Functional MRI research has demonstrated distinct brain activation patterns across different OCD subgroups [35, 36]. If similar subtype-specific patterns can be detected using EEG, it could pave the way for developing personalized neurofeedback and TES protocols tailored to individual symptom profiles.

None of the reviewed articles addressed OCD subtypes, such as symmetry or contamination obsessions, representing a notable research gap. Future studies should investigate whether different OCD subtypes exhibit distinct EEG patterns. Only one study categorized OCD patients based on high and low anxiety levels, but this classification did not align with DSM-defined subtypes. Seven studies (63%) reported details about the specific OCD populations included. Three studies (27%) did not mention OCD subtypes, and one study omitted demographic information entirely. Studies with smaller sample sizes. For example, Mukherjee et al. with 46 patients often reported unusually high classification accuracy (up to 100%), while those with larger samples such as Farhad et al. (2024). with 86 patients tended to report more moderate and stable accuracy rates (ranging from 75 to 90%).

### EEG electrode montage and frequency bands involved

Eight studies used the standard 10–20 EEG montage, while two employed the higher-resolution 10–10 montage. One study did not specify the montage used. A significant research gap was the lack of information on reference electrodes: six studies (~ 54%) did not report which reference was used. This omission hinders reproducibility and limits the ability of other researchers to replicate or build upon these findings.

The classic four EEG bands (delta, theta, alpha, and beta) were analyzed in 54% of the studies. Three studies (27%) focused exclusively on these bands, while another three (27%) also included high beta and gamma frequencies. Two studies utilized raw EEG signals, and two did not specify which frequency bands were used. One study employed EEG microstate analysis. Notably, both studies that used raw signals applied deep learning techniques and required larger sample sizes, reflecting the increased data demands of such approaches.

Most studies focused on the resting-state condition, particularly with eyes closed. Approximately 81% (*n* = 9) of the articles used resting-state EEG; of these, seven studies employed the eyes-closed condition. One study combined resting-state and task-based recordings, while another did not specify the condition used. The eyes-closed resting-state may help minimize visual processing biases; however, it is also associated with increased alpha activity, which could influence the interpretation of EEG findings in OCD research.

### Data preprocessing

Bandpass filtering and Independent Component Analysis (ICA) were among the most commonly used preprocessing techniques. Of the 11 studies, five applied bandpass filtering, and three used ICA. Reported classification accuracies in ICA-based studies ranged from 68 to 91%. However, due to variability in machine learning models and classification objectives, it is difficult to isolate the specific impact of ICA on performance. Two studies did not report any preprocessing steps. While proper preprocessing is generally believed to enhance machine learning accuracy, the inconsistency in methods across studies prevents firm conclusions. The adoption of standardized preprocessing guidelines, such as those proposed by COBIDAS-EEG, is recommended to improve comparability and reproducibility.

As shown in Table 7, earlier studies tend to report lower classification accuracies, while more recent publications show higher performance. One likely explanation is the ongoing development of machine learning techniques within psychiatric research. Over time, the field has moved from using relatively simple models to employing more advanced methods, such as deep learning and combinations of multiple algorithms. Alongside these advancements, there have also been notable improvements in EEG data processing. For example, in how noise and artifacts are removed, interpolation of channels in clinical data, and how relevant features are extracted from the signals.

**Table 7:** Summary of reported classification accuracies for OCD disorders using EEG-Based machine learning models

Study	Model	evaluation metrics	OCD accuracy (%)	overall accuracy (%)	Explain
Ahmed et al., 2024	ANN	Accuracy	96.83	87.00	-
	KNN	Accuracy	94.70	85.00	-
	LSTM	Accuracy	93.65	86.00	-
	Bi-LSTM	Accuracy	93.12	87.00	-
	CNN-LSTM	Accuracy	92.06	86.00	-
Basak et al., 2023	LSTM	Precision/Recall/F1	95/100/97	89.85	Validation
Emre et al., 2023	C5.0 (Imbalanced)	Balanced Accuracy	-	70.60	Sensitivity = 42.9
	SVM (Oversampling)	Balanced Accuracy	-	90.30	Specificity = 95.0
	RF (Undersampling)	Balanced Accuracy	-	89.50	-
Erguzel et al., 2015	ANN	Accuracy	63.29	-	-
	KNN	Accuracy	56.96	-	-
	SVM	Accuracy	67.08	-	-
	Naive Bayes (NB)	Accuracy	56.96	-	-
	IACO-SVM	Accuracy	81.04	-	-
Farhad et al., 2024	1DCNN-LSTM (CV)	Accuracy	90.88	-	Cross-Validation
	1DCNN-LSTM (EV)	Accuracy	75.00	-	External Validation
	1DCNN-GRU (CV)	Accuracy	85.91	-	-
	1DCNN-GRU (EV)	Accuracy	75.00	-	-
Gour et al., 2023	Transformer + CCE	Accuracy	-	63.21	Eyes Open
	Transformer + FL	Accuracy	-	68.49	-
Ma et al., 2025	XGBoost (Subgroups)	Test AUC	-	77.13	-
Mukherjee et al., 2024	PsyNet	Accuracy	100.00	95.97	-
Park et al., 2021	Elastic Net (Gamma)	Accuracy	74.52	87.59	-
	SVM	Accuracy	-	86.02	-
	RF	Accuracy	-	87.18	-
Ren et al., 2024	SVM (Time Features)	Accuracy	75.86	-	-
	LR (Nonlinear)	Accuracy	85.00	-	-
	GNB	Accuracy	80.00	-	-
Verma et al., 2024	CNN-GRU (Beta Band)	Accuracy	90.00	90–96	-

Another factor that may contribute to the improved results is the availability of more powerful computing tools and, in some cases, larger and better-quality datasets. These conditions make it easier to train and test models more effectively. In addition, some recent studies have made an effort to incorporate clinical variables, like different OCD symptom dimensions or treatment response profiles, which may help improve model relevance and accuracy. At the same time, it is worth being cautious. Not all increases in accuracy reflect genuine methodological progress, some may result from small sample sizes, lack of external validation, or overly optimistic performance metrics. These issues should be taken into account when interpreting trends over time.

### Accuracy reporting

**Fig. 3:**
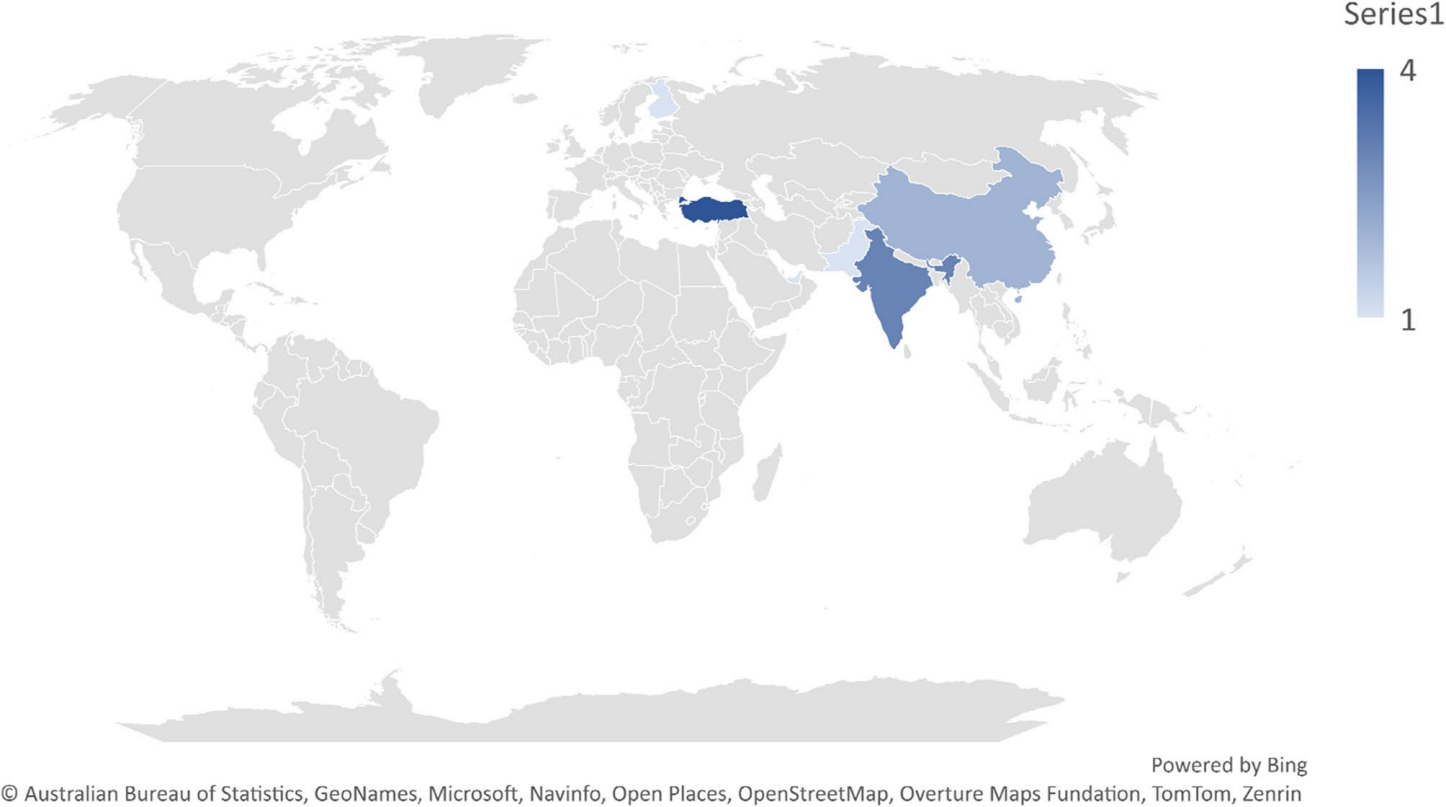
Geographical Distribution of Studies

Reviewed studies employed various methods to report classification accuracy (cf., Table 7), making it difficult to identify the best-performing model. Differences in validation approaches significantly influenced reported accuracies. For example, a 1D CNN-LSTM model achieved 90.88% accuracy during cross-validation but dropped to 75% when tested on an external dataset. This highlights the need for standardized accuracy metrics and validation protocols. Since most studies reported overall accuracy, we used this measure for comparison (cf., Fig. 3). Due to the generally small sample sizes, analyzing additional performance metrics was not feasible. In general, deep learning models tended to outperform classical machine learning approaches.

### Machine learning models

**Fig. 4:**
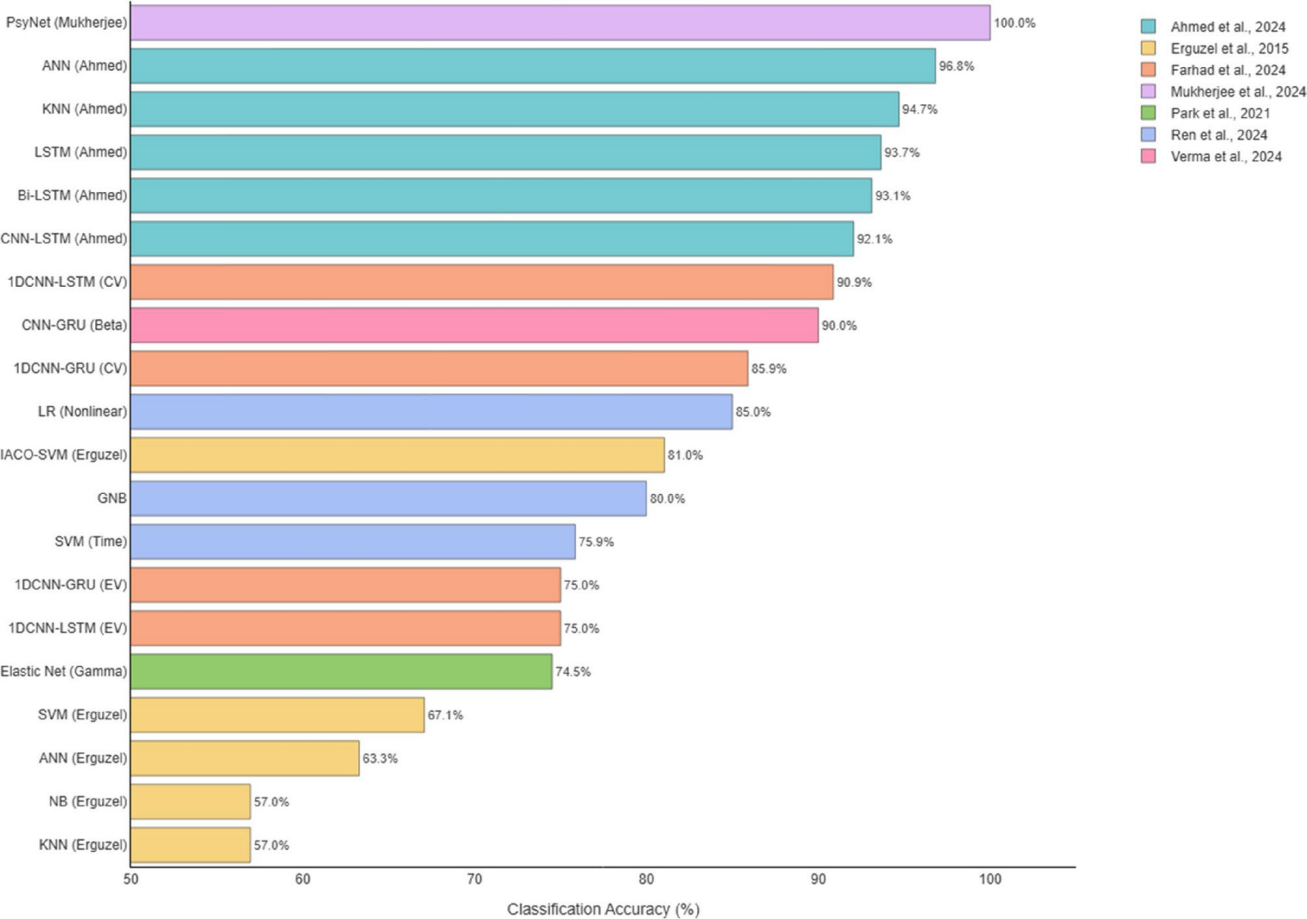
Distribution of Reported Classification Accuracies for OCD Groups - Although accuracy is a common measure in studies, the difference in comparison groups (for example, healthy control group versus clinical control group) creates big differences. Thus, we must be coutious when comparing performance between studies

**Fig. 5:**
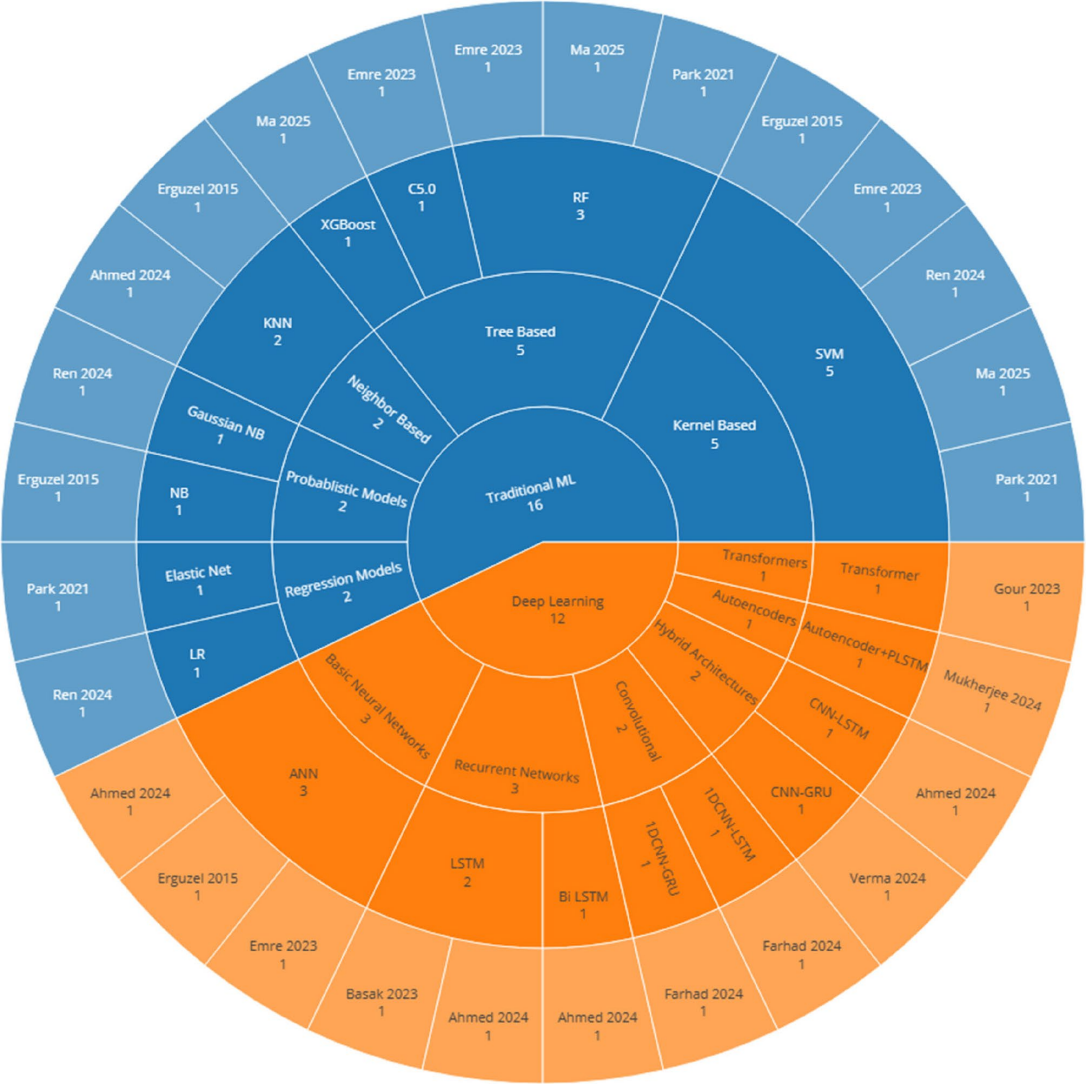
Hierarchy of Machine Learning Methods in OCD-EEG Studies

According to Fig. 4, most articles used classical machine learning models. The most common model was SVM (used in 5 articles). Among deep learning models, ANN appeared in 3 articles. Tree-based models like RF, C5.0, and XGBoost were also common (5 articles) (Fig. 5).

### Interpretability methods

None of the reviewed studies employed modern interpretability methods such as SHAP or LIME. The authors recommend that future research incorporate these techniques to enhance the transparency of machine learning models, facilitating their translation into clinical tools like neurofeedback or electrode placement guidance for brain stimulation. While three studies attempted to interpret EEG features using statistical tests which is a positive step, these approaches are insufficient on their own to fully elucidate model decision-making.

### Methodological comparison across studies using a shared dataset

Four studies reviewed in this paper (Park et al., 2021; Ahmed et al., 2024; Basak et al., 2023; and Mukherjee et al., 2024) all used the same dataset from Park 2021. This shared data gives a special chance to compare the methods used in each study. Because the data is the same, we can better see how model design, data processing, and validation affect the results. The accuracy reported in these studies is very different, from 74.52 to 100%. This shows that the choice of model and how complex it is can change the results a lot. For example, Mukherjee et al. used a deep model with Autoencoder and PLSTM and reported 100% accuracy. But they only used internal validation (cross-validation), and did not test the model on new data. So, this high result may not be real in practice and could mean overfitting. On the other hand, the simpler model Elastic Net used by Park et al. showed 74.52% accuracy, but it may generalize better because it is simple and clear.

To study how data preprocessing affects model results, we tried to find the same model in two papers: Ahmed and Basak. Both used LSTM models, but with different preprocessing. Ahmed used filtering, standardization, and coding. Basak used downsampling and moving average. But they used different ways to report results (Ahmed used direct accuracy, Basak used F1-Score), and they used different validation methods (cross-validation vs. train/test split). This made it hard to compare their results fairly. Also, Mukherjee et al. did not give information about preprocessing, so we could not include it in our data table. Because of these problems, we cannot clearly say how preprocessing affects model results in these studies.

All four studies used internal validation. None of them tested their models on new or outside data. Also, no study said clearly if their data split was based on subjects or just on EEG segments (epochs). This is important, because if data from the same person is in both training and testing, the result may look better than it really is. Without outside testing and clear reporting, it is hard to compare the models. We suggest that future studies use external validation and split data by subject, so we can understand how well models work in the real world. Using the same dataset across studies can also help us compare methods better, and this should be done more in future reviews.

## Discussion

In this systematic review, we aimed to review studies that have been attempted to classify OCD compared to healthy or other psychiatric and neurological conditions using EEG and with an application of machine learning and deep learning techniques. In our review, we witnessed that studies usually do not focus on one or two disorders but focus on large categories of disorders. It is suggested that e.g., journals, as topic planners for special issues, should encourage topics with more clinical use while respecting basic research, like studies that are more interpretable, not just research with higher accuracy or more disorders. These types of reports, only with limited information provided lead to misundersatidng and misinterpretations given the complexity if the disorder and associated morbidities. In recent years, with an advancement of AI, more researchers are interested to distinguish neuropsychiatric conditions against healthy condition and to further develop precision medicine and individualise treatments. Although the small number of studies and the high heterogeneity of extracted data limit quantitative analysis, this variability highlights a critical issue: the lack of internationally standardized research designs and methodological perspectives to efficiently investigate the differences in detection of mental disorders and to predict the onset or monitirng the treatment such as phramocotherapy. The absence of such standards hampers the reproducibility of findings. In this section, we first discuss the current state of the field based on the reviewed studies, followed by future directions where we propose a practical framework. This framework aims to address existing challenges, improve study repeatability, and ultimately enable meaningful meta-analyses.

### Current status

#### Accuracy paradox and high heterogeneity among the included studies

While high accuracy in a computational model would first be thought of as an achievement, the problem is that extremely high accuracy levels can occur from overfitting or from an oversmall sample. In the study by Mukherjee et al. (2024), the PsyNet model with Autoencoder + PLSTM architecture was 100% accurate, but since the sample size was very low (only 46 participants) and there was no external validation, the genralisability of results must be dealt with cautious. In studies using external validation — for example, Farhad et al., 2024 — the accuracy decreased from 90.88% in cross-validation to 75% in external validation, clearly showing that how accuracy was reported had a great impact on the percentage reported. Future work, therefore, should focus on comparing various models and evaluate the extent to which higher accuracies are observed, while also achieving reproducibility, uniform reporting of accuracy, representative sample size, and transparency of method should be considered in order to provide the solid groundwork for progressing EEG-ML as useful clinical tools. More specifically, considering that OCD is a heterogenous neuropsychiatric disorder with other morbidities or variety of symptoms severity, require more indepth investigations by including demographic information of patients and aged matched healthy individuals, medication stutus, years of illness, types of treatments and behavioral and cognitive components associated with both healthy and OCD patients. These considerations would better contribute to our understanding about the disorder and neurobehavioral and cognitive dimensions impaired due to the symptoms and the history of individuals. This further enables clinicians to develop an evidenced-based therapeutic models for the OCD patients depending on their age and with higher precision. A key contributor to the high heterogeneity observed across EEG-based machine learning studies in OCD is the significant variation in clinical, methodological, and technical approaches. Clinically, participant characteristics differ widely between studies. These include variations in symptom severity, diagnostic procedures, comorbidity profiles, and treatment history. While some studies used structured diagnostic interviews such as the MINI or Y-BOCS, others relied on broader psychiatric evaluations or failed to specify their diagnostic criteria. Treatment status also varied: some studies included drug-naïve participants, whereas others examined individuals undergoing pharmacological interventions such as SSRIs or antipsychotics—factors known to influence neural activity and EEG signatures.

From a technical perspective, there is a lack of standardization in EEG acquisition and processing protocols. Studies differed in their choice of electrode montages, frequency bands of interest, filtering techniques, and resting-state conditions (e.g., eyes open vs. eyes closed). These discrepancies inevitably affect the recorded signal characteristics and complicate cross-study comparisons. Additionally, preprocessing pipelines and feature extraction strategies were inconsistent, ranging from handcrafted spectral features to deep learning-based representations. Machine learning models also varied substantially—not only in algorithmic choice (e.g., SVM, ANN, LSTM, CNN-GRU, XGBoost), but also in how model performance was evaluated (e.g., accuracy, F1 score, AUC, balanced accuracy), and whether the evaluation used cross-validation, external validation, or subgroup analysis.

Performance outcomes reflect this diversity. For instance, recent studies such as Mukherjee et al. (2024) and Ahmed et al. (2024) reported remarkably high classification accuracies (up to 100% and 96.83%, respectively), while others like Gour et al. (2023) and Erguzel et al. (2015) reported more modest results (e.g., 63.21% and 56.96%). These discrepancies cannot be solely attributed to model quality but also reflect underlying variability in dataset composition, preprocessing rigor, and evaluation standards. Studies with external validation, such as Farhad et al. (2024), often report lower accuracies (e.g., 75.00%), underscoring the challenges of generalizability in real-world settings.

Overall, the primary source of heterogeneity seems to lie in the lack of standardization in both clinical characterization and EEG processing workflows. Without consistent approaches to defining OCD subtypes, accounting for comorbidities, or applying harmonized preprocessing and modeling techniques, it remains difficult to make meaningful comparisons or draw robust conclusions across studies. Future research should prioritize the adoption of standardized protocols, greater transparency in reporting methodological details, and inclusion of metadata related to clinical profiles and signal processing steps. These measures are critical for improving reproducibility and advancing the clinical utility of EEG-based models in OCD classification.”

#### Continued preference for traditional models and trends in deep learning and hybrid architectures

Despite the increasing attention toward modern machine learning techniques, traditional models still dominate EEG-based classification studies. This ongoing reliance can be attributed to several practical reasons: classical models such as SVM, and tree-based algorithms are computationally efficient, require less training data, and are more familiar to researchers. SVMs, in particular, have remained widely used due to their ability to handle high-dimensional feature spaces and employ nonlinear kernels for detecting complex patterns. Similarly, tree-based models have remained popular because they offer built-in dimensionality reduction through ensemble strategies like bagging, while also providing interpretable outputs through feature importance rankings.

While deep learning models were less frequently used in earlier studies, there has been a noticeable increase in their adoption in more recent literature (2023–2025). This shift reflects a gradual movement toward more sophisticated modeling techniques that can better capture the dynamic and high-dimensional nature of EEG signals. Among these, LSTM networks and their variants such as Bi-directional LSTM (Bi-LSTM) have emerged as the most commonly applied architectures, representing 30.8% of all deep learning approaches observed. These models are particularly well-suited for EEG data, as they are designed to model sequential dependencies and preserve important temporal information over time.

The suitability of LSTM-based models is especially relevant in the context of OCD research, where datasets tend to be relatively small. Unlike many deep learning architectures that demand large-scale data, LSTM models have shown greater resilience in smaller datasets, making them a practical and powerful tool for identifying temporal dynamics and subtle neurophysiological patterns often missed by traditional approaches. Their increasing usage signals a growing confidence in deep learning’s potential to enhance precision and interpretability in EEG-based OCD classification. Importantly, LSTMs are also more robust when dealing with small datasets—a recurring challenge in OCD-related EEG research. In machine learning, a common rule of thumb suggests that at least 10 samples per feature are needed to avoid overfitting. Given that EEG studies typically extract hundreds of features—e.g., from 19 electrodes across 5 frequency bands—a dataset with fewer than 100 participants is at high risk of overfitting. Yet, only seven OCD studies in this domain explicitly report sample sizes, and none include more than 95 OCD patients. This further justifies the use of LSTM-based models, which tend to perform better under such constraints compared to other deep learning architectures. As datasets grow and become more standardized, this trend toward deep learning is likely to accelerate.

Additionally, choosing between using raw signals or extracted features for EEG-ML in OCD is a complex problem. To our knowledge, it is not possible to say clearly if raw signals or extracted features are better. Raw signals, especially when given to LSTM or Transformer, may help models understand time series, sequences, and nonlinear features but may also include more noise. Using extracted features can reduce noise but hide time sequence data. We think both views are valuable, but it is recommended that if using extracted features, high beta and gamma bands should be included, because research has shown these bands are valuable for OCD diagnosis [10, 37–39]. Studies not analyzing these bands may miss key information, especially changes in e.g., associated with clinicl symptoms, monitoring medication effect and associated improvement.

#### Future directions: building toward reproducibility and clinical translation

To move EEG-based OCD research closer to clinical applications, future efforts must focus on consistency, transparency, and broader population relevance. One major challenge in this field is the lack of standardization, which makes it difficult to compare findings across studies or replicate results. Without consistency in methods and clarity in interpretation, promising discoveries may struggle to translate into clinical practice.

A notable observation from current literature is the geographic concentration of studies, with a majority carried out in Asian populations. While valuable, this regional focus may limit how well the findings apply to more diverse global populations. Expanding the use of publicly accessible EEG datasets that include a broader cultural and demographic range could help overcome this limitation.

Establishing a large-scale, open-access EEG repository with standardized protocols and data from varied populations would be a key step forward. Such a resource would not only strengthen reproducibility but also support more robust cross-study comparisons. This approach mirrors the success of the Human Connectome Project in the domain of fMRI, and serves as a model worth adapting for EEG research. Additionally, open datasets can help reduce the financial burden on individual research teams, foster collaboration, and accelerate the pace of innovation. They also promote transparency and allow the community to test and validate findings on independent data an essential requirement for clinical acceptance.

In sum, creating shared frameworks and accessible resources will be crucial for ensuring that findings in this field are not only scientifically sound but also relevant and usable in real-world clinical settings. Despite this need, an ethical framework need to be proposed by the scientific community due to the neurobiological datasets shared from human patients. A standardized broader framework that if not globally, but continent-wise is important. This would enable researchers to collaboratively share and investigate various dimensions of data based on their expertise and advance the field in a more efficient and constructive manner while respecting ethical concerns and regional and international data privacy law.

#### Data preprocessing

The reviewed literature demonstrates considerable variability in EEG preprocessing methods. We recommend the use of the COBIDAS-MEEG toolbox, which includes the following standardized steps: (1) detection and removal of bad channels, (2) artifact cleaning using techniques such as Independent Component Analysis (ICA) to remove eye and cardiac artifacts, (3) high-pass and low-pass filtering, (4) signal segmentation, (5) baseline correction, (6) transformation of electrode references, and (7) spectral transformation for frequency analysis. We also recommend furture research to test the differences across the same datasets when bad channels are interpolated and not, as in our opinion, noises might be part of the clinical symtpms. To ensure reproducibility, each step should be performed using consistent parameters and reported in exhaustive detail. Strict adherence to this standardized protocol will enhance methodological comparability and repeatability across studies.

#### Reporting accuracy

The reviewed studies exhibit considerable variability in how accuracy is reported. To address this issue, we recommend adhering to the TRIPOD guidelines (Transparent Reporting of a Multivariable Prediction Model) and encouraging the publication of full model training code, either within the paper or on platforms such as GitHub. Combining TRIPOD with the COBIDAS-MEEG standards for EEG preprocessing can establish a robust scientific foundation for the standardization of EEG-based machine learning research. This harmonization will facilitate more meaningful comparisons across studies and accelerate clinical adoption of predictive models. We further propose that international consortia should develop a specialized TRIPOD-EEG extension to better suit the unique aspects of EEG-based machine learning studies.

#### Model interpretation

Many models have been treated as “black boxes” without sufficient interpretation. Identifying which biomarkers, electrodes, or EEG waveforms are critical for OCD diagnosis and it is essential not only for accurate diagnosis but also for guiding treatment and precision medicine. Currently, interpretability is lacking, which undermines clinical trust in these models and limits the development of applications such as tES electrode placement and neurobiofeedback using feedback loops. As a result, most studies focused solely on binary diagnosis, restricting their potential impact on treatment strategies. Successful examples, such as FDA-approved neurofeedback devices for anxiety, demonstrate that EEG biomarkers can underpin non-invasive diagnostic and therapeutic tools.

While we encourage the integration of SHAP and LIME to enhance model transparency, it is important to recognize the practical considerations when applying these methods to EEG data. First, the effectiveness of these techniques is heavily dependent on the quality and relevance of input features. EEG data, often high-dimensional and noisy, can pose challenges in feature extraction, potentially reducing the interpretability of model outputs. Second, SHAP and LIME assume some degree of model agnosticism or structural compatibility—certain deep learning architectures, especially those working directly on raw EEG time-series data, may require preprocessing steps such as dimensionality reduction or segmentation to produce meaningful explanations. Third, interpreting the resulting importance scores in a neurophysiological context is non-trivial, as EEG features may not map directly to intuitive biomarkers without further domain knowledge or validation. Thus, while SHAP and LIME hold promise for improving transparency and guiding clinical applications (e.g., neurofeedback electrode placement or TES targeting), their implementation must be carefully tailored to the data type and clinical goals. Future studies should aim to validate the interpretability outputs with domain experts and explore hybrid strategies combining model-specific interpretability with domain-relevant visualization tools.

Such interpretability tools could serve as a bridge between machine learning model outputs and actionable clinical insights, such as identifying relevant biomarkers for individualized tES targeting or optimizing neurofeedback protocols. Their integration could significantly advance trust and usability in clinical environments, provided their methodological constraints are transparently addressed.

Overall, the lack of a clear conceptual and methodological connection between the reviewed findings and the specific symptoms of OCD makes it challenging to interpret the results in a way that is clinically useful. This gap seems to reflect the nature of the research questions often asked in these studies, which are frequently shaped by researchers from technical or engineering backgrounds. As a result, many of the studies tend to focus heavily on algorithmic or signal processing aspects, without anchoring their analyses in established clinical knowledge about OCD or in its underlying neurobiology. For example, few studies link their EEG findings to known patterns in OCD, such as altered theta or alpha activity, or consider how these patterns might differ across symptom types like contamination fears or compulsive checking. Similarly, the possibility of using EEG markers to predict responses to treatments such as SSRIs or cognitive behavioural therapy is not addressed. Without incorporating these clinical aspects, the research risks making broad, generalized claims that may not translate meaningfully to OCD. This lack of integration reduces the potential of EEG-ML methods to inform clinical practice or deepen understanding of OCD specifically. Going forward, studies, clinicians and patients involved would benefit from incorporating clinical variables such as symptom subtype, severity, or treatment history into their models and analyses. Aligning methodological development with clinical and theoretical knowledge about OCD could help the field move toward more targeted and informative findings, rather than remaining at the level of general critiques or technical demonstrations.

#### From challenge to vision

**Fig. 6:**
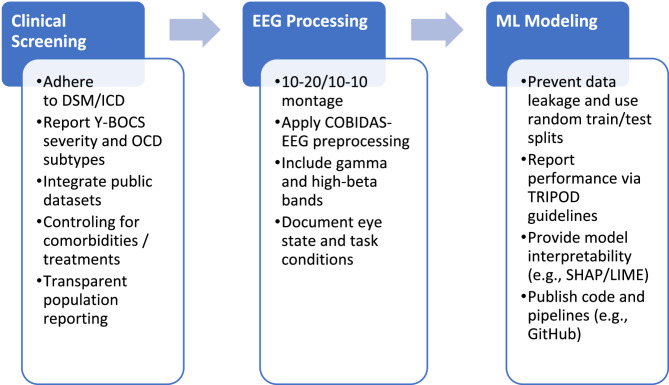
Three-stage standardization framework to reduce methodological heterogeneity and reduce research gap in EEG-based machine learning studies for OCD

The greatest challenge identified in this review is the significant heterogeneity across the current literature, which complicates the identification of common features. To address this, we propose a three-stage framework (cf., Fig. 6) designed to guide researchers toward greater standardization and consistency by adhering to established protocols and addressing existing research gaps.

#### Towards clinical translation

To move EEG-based machine learning from research to clinical settings, three translation gaps must be filled:

1. Regulatory readiness: Models must meet repeatability criteria in Fig. 6 to be homogeneous and FDA-reviewable.
2. Interpretability integration: Combine SHAP/LIME with models for better electrode placement in neurofeedback and TES, and better analysis of gamma and high beta waves.
3. Comorbidity management: Develop hybrid EEG-fMRI biomarkers to differentiate OCD from e.g., Depression/ADHD. Pilot studies using portable EEG headsets can test real-world feasibility.

### Limitations

Despite conducting a thorough search across four major databases (PubMed, Scopus, IEEE, and Web of Science), and adhering to PRISMA guidelines, only 11 studies met our inclusion criteria. Considerable heterogeneity was observed across multiple aspects, including EEG recording protocols, preprocessing techniques, feature extraction methods, machine learning algorithms, and accuracy reporting. These challenges led to two primary conclusions: first, there is a critical need for standardized protocols and frameworks for EEG data acquisition and machine learning reporting; second, significant research gaps exist regarding model interpretability and the differentiation of OCD subtypes. Recognizing these limitations provides valuable insights that can guide future research toward greater reproducibility, enable more robust meta-analyses, and accelerate the translation of EEG-based machine learning approaches from experimental studies to clinical applications in OCD.

#### Future computationl and clinical direction

While the reviewed studies suggested promising avenues for using EEG and machine learning models in classifying OCD, the evidence base remains preliminary. Most included studies suffer from small sample sizes, lack of consistent external validation, and limited or absent interpretability methods. Therefore, translating these tools into clinical practice including regulatory milestones such as FDA approval or application in neurofeedback and tES targeting remains a long-term goal rather than an immediate prospect. Clinical utility is contingent on the development of standardized protocols, consistent reporting practices, and rigorous validation frameworks. Until these conditions are met, findings should be interpreted cautiously, especially when reported in terms of diagnostic or therapeutic utility such as tES and neuro-biofeedback interventions.

We suggest future studies to further consider following points during investigations:

As outlined in clinical diagnostic guidelines, OCD frequently co-occurs with a range of other neurodevelopmental and psychiatric conditions. When building computational models for OCD diagnosis, it’s important to account for this overlap. Brain activity patterns in individuals with OCD can be influenced by comorbidities such as anxiety disorders, ADHD, PTSD, tic disorders, and mood disturbances. These variations, shaped by the diverse clinical profiles seen in OCD, must be carefully considered to ensure that diagnostic models are accurate and generalizable.

Variations in OCD symptoms, treatment type, timing, and response to the clinical efforts also present key considerations for modeling. Pharmaceutical interventions, the stage of illness (e.g., early onset, chronic OCD), timing of symptom presentation, and the trajectory of therapeutic response all influence brain dynamics in meaningful ways. In particular, individuals with treatment-resistant OCD—an underrepresented group in many studies—may show distinct neurophysiological patterns. Including data from such cases can enhance predictive models and help tailor interventions such as cognitive-behavioral therapy, pharmacotherapy, neurofeedback, or neuromodulation techniques like transcranial magnetic stimulation.

Demographic variables, such as age and gender, also play a significant role in shaping OCD’s neural profile. For example, pediatric-onset OCD may present differently from adult-onset cases, and symptom expression often varies between males and females. Diagnostic models should be refined by integrating such demographic distinctions along with clinical factors like symptom severity, compulsivity levels, insight, and the presence of comorbid behaviors such as avoidance or sleep disruption. These parameters help create a more nuanced and individualized diagnostic approach.

To better differentiate OCD-related brain activity from healthy patterns, statistical analysis of extracted features should be complemented by more advanced signal processing techniques. Methods such as power spectral density (PSD) analysis and functional connectivity mapping can uncover subtle but meaningful distinctions in resting-state activity. Focusing on specific frequency bands and relevant brain regions those found to show altered communication in OCD can improve classification accuracy and strengthen the diagnostic relevance of these computational tools.

In addition, some studies in this review reported very high accuracies or made claims regarding clinical applicability without adequate supporting evidence. For instance, the use of terms like “diagnostic tool” or “clinical prediction” was sometimes made in the absence of external validation, interpretability analysis, or outcome-based evaluation. To improve scientific transparency and prevent overinterpretation:

- Future authors should clearly state whether their findings are exploratory or validated.
- Claims about clinical utility should be qualified unless supported by prospective trials, larger sample sizes, or real-world evaluations.
- Interpretability tools (e.g., SHAP, Grad-CAM) should accompany performance metrics to support clinical translation.
- Studies should avoid equating high cross-validation accuracy with diagnostic readiness.

Finally, dimensionality reduction and advanced modeling techniques offer promising pathways to uncover deeper patterns within the data. Techniques like t-distributed stochastic neighbor embedding (t-SNE) can be used to explore whether neural data clusters by OCD symptom severity or subtype. Similarly, using architectures like autoencoders, transformers, or unsupervised learning methods can help reveal hidden structure in EEG data, enhance model interpretability, and support the development of more robust, data-driven diagnostic frameworks for OCD.

## Conclusion

This systematic review highlights the emerging yet highly heterogeneous field of EEG-based machine learning for classifying OCD. Traditional machine learning models such as SVM and Random Forest have been commonly used, while more recent studies increasingly apply deep learning architectures like LSTM and CNN-LSTM. Reported classification accuracies varied widely, with studies employing external validation demonstrating more reliable results. These findings underscore the urgent need for standardized protocols in EEG data acquisition, preprocessing, model reporting, and interpretability. Our proposed three-step standardization framework offers a practical roadmap to enhance reproducibility and lay the foundation for future meta-analyses. While EEG-based machine learning holds potential for aiding OCD diagnosis, the current state of the field does not yet support clinical deployment. Future research should prioritize large, well-characterized samples, standardized protocols, interpretability tools, and outcome validation to move toward genuine clinical readiness.

## Data Availability

No datasets were generated or analysed during the current study.

## Abbreviations

SCZ: Schizophrenia
MDD: Depression disorder
BD: Bipolar disorder
PD: Panic disorder
SAD: Social anxiety disorder
OCD: Obsessive-compulsive disorder
AUD: Alcohol use disorder
BA: Behavioral addiction
PTSD: Post-traumatic stress disorder
ASD: Acute stress disorder
AD: Adaptation disorder
SUD: Substance use disorder
ADHD: Attention-deficit/hyperactivity disorder
OUD: Opiate use adective
HC: Healthy control
TTM: Trichotillomania
SMC: Subjective memory complaints
